# Comparison of Furlow’s Double Opposing Z-plasty and Straight-Line Intravelar Veloplasty as Methods of Cleft Palate Repair

**DOI:** 10.7759/cureus.52897

**Published:** 2024-01-25

**Authors:** Ravikumar Mahajan, Ankush Tambotra, Harish Ghildiyal, Mahipal Singh, Thakur Thussu, Abhishek Bhamre, Krishnan Srinivasan

**Affiliations:** 1 Plastic and Reconstructive Surgery, Amandeep Hospital, Amritsar, IND; 2 Plastic Surgery, Amandeep Hospital, Amritsar, IND

**Keywords:** soft palate, fistula, straight-line intravelarveloplasty, furlow’s z-plasty, cleft lip and palate

## Abstract

Background

One of the common craniofacial abnormalities is cleft lip and palate. Various surgical procedures have been employed to repair the cleft. However, immediate post-operative complications and formation of palatal fistula post surgery are common in surgical procedures. The study aims to compare the fistula rate, soft palate lengthening, and immediate complications of cleft palate repair of Furlow’s Z-plasty and straight-line intravelar veloplasty techniques.

Method

Fifty Patients with isolated or unilateral cleft palate and lip with age between 9-18 months were randomly divided into two groups. One group underwent Furlow’s Z-plasty while the other underwent straight-line intravelar veloplasty procedures. Post surgery, after discharge, the patients were followed up at 2 weeks, 1 month, and 3 months intervals. Immediate post-operative complications and fistula formation rate were compared along with other parameters like fistula width, duration of the procedure, intra-operative soft palate length, etc.

Results

Straight-line procedures took less time as compared to Furlow’s Z-plasty. Bleeding (N=2, 8%) and dehiscence (N=1, 4%) of the wound were the immediate post-operative complications found in the straight-line group. Bleeding was also present in the Furlow's group (N=1, 4%). At 1-month follow-up, in the straight-line group (N=3, 12%) patients had fistula while (N=2, 8%) had minimal nasal regurgitation of liquids when compared to the Furlow's group. At 3-month follow-up, patients in the straight-line procedure group (three out of N=25, 12%) exhibited fistula, whereas in the Furlow's group, fistula occurrence was observed in one out of N=25 participants (4%). Intra-operative soft palate lengthening was 6.44 ± 0.768 mm and 1.64 ± 0.952 mm in the Furlow and straight-line groups, respectively.

Conclusion

Furlow's Z-plasty was observed to be the better surgical procedure for cleft repair as it had low immediate post-operative complications, and fistula development and had higher intra-operative soft palate lengthening.

## Introduction

Cleft lip and palate are the most common congenital craniofacial anomalies [1]. The overall prevalence of orofacial clefts is estimated to be approximately 1 in 700 live births, accounting for nearly one-half of all craniofacial anomalies [2]. Cleft palate (CP) presence affects both aesthetic and functional abilities of the patients in their social interactions, particularly their ability to communicate effectively and their facial appearance with or without the involvement of the lip. Mid-facial skeletal growth may also be affected by the surgical repair of the palate. The treatment plan mainly focuses on two areas: speech development and facial growth while minimizing the incidence of palatal fistula. Speech development is paramount in the appropriate management of cleft palate. The goal of palate repair is to separate the oral and nasal cavities and create a competent velopharyngeal valve for swallowing and speech while preserving midface growth and development of functional occlusion [3]. Of the many variables that influence outcome after CP repair, the most important are probably the timing and technique of palatoplasty.

Numerous surgical approaches and adaptations, such as those involving straight-line procedures like straight-line intravelar veloplasty and Veau-Wardill-Kilner pushback palatoplasty, along with Furlow's Z-plasty, have been proposed to enhance the functional and aesthetic outcomes of cleft palate surgery [4-6]. The major issue with these surgical interventions is the development of palatal fistulas, ranging from asymptomatic holes to large communications between the oral and nasal cavities that cause speech problems, nasal regurgitation, and hygiene difficulties. If symptomatic, fistulas can be surgically corrected with local mucosal flaps, loco-regional and distinct flaps [7]. Fistula rates following primary palatoplasty have historically been reported at over 60 percent and in recent literature have ranged from 2.4 to 35 percent. Factors affecting fistula formation include the cleft anatomy, type of repair, cleft width, cleft width to total palatal width ratio, and the surgeon’s experience.

In the current study, we primarily compare the fistula rate and immediate complications of cleft palate repair of Furlow's double opposing Z-plasty (Furlow’s Z-plasty) and straight-line intravelar veloplasty techniques, as speech evaluation needs longer follow-up.

## Materials and methods

This prospective cohort study was conducted after receiving approval (ECR/692/Inst/PB/2014/RR-20) from the Amandeep Hospital Ethics Committee during the period between February 2016 to March 2018.

Patient selection

Fifty patients with isolated cleft palate and unilateral cleft lip and palate, within the age range of 9-18 months were selected for the study. Patients with ages < 9 months and > 18 months, patients with syndromes, patients who were medically unfit for the surgery, had bilateral cleft lip and palate (due to a smaller number of cases) or submucous cleft palate or otogenic intra-cranial complications, children who had developmental delays or were not gaining weight properly as per the age, and children whose consent was refused by parents were excluded from the study. Prior history and general physical and systemic examination, examination of the oral cavity, radiological investigations, haemogram, and pre-anaesthesia check-up of the patients were performed before surgery.

Operative procedure

The All the patients were randomly distributed by simple randomization (i.e., flipping a coin) into two groups of 25 each for comparison between Furlow’s and straight-line intravelar veloplasty. General anaesthesia was used for all the cases.

Furlow's Double Opposing Z-Plasty

**Figure 1:**
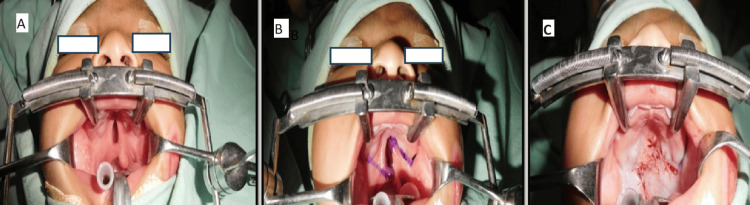
Representative images showing Furlow’s Z-plasty A) Incomplete cleft palate before surgery; B) marking for Furlow’s Z-plasty before the surgery; C) cleft closure after Furlow’s Z-plasty.

The principle of Furlow's Z-plasty involves transposition rather than transection of the palatal muscles. The palatal muscle was elevated as part of the posterior-based flap. The nasal Z-plasty was made as the mirror image of the oral layer. The lateral limbs of the oral Z-plasty ended over the hamuli. The posterior-based flap on the left side had an angle of about 60 degrees. The lateral limb of the anteriorly based flap on the right side had an angle of almost 90 degrees. The left cleft margin was incised first and the mucomuscular flaps were raised without any lateral relaxing incisions (Figure 1).

Straight-Line Procedures

**Figure 2:**
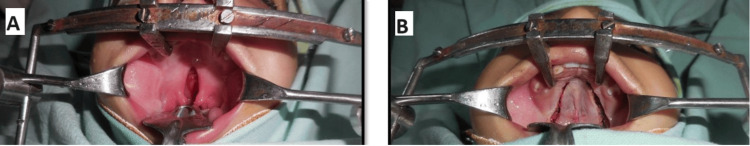
Representative images showing straight-line intravelar veloplasty A) Incomplete cleft palate before surgery; B) Cleft closure after straight-line intravelar veloplasty.

The Von Langenbeck palatoplasty is a straight-line procedure that involves relaxing incisions along the lateral edge of the hard palate, starting anteriorly near the palatomaxillary suture line, running posteriorly just medial to the alveolar ridge, and ending lateral to the hamulus, about 1 cm posterior to the greater tuberosity of the alveolus. The mucosa along the edges of the cleft was also incised. Mucoperiosteum was raised from the oral surface of the hard palate, while the two neurovascular pedicles, i.e., the greater palatine pedicle posteriorly and the incisive pedicle anteriorly were preserved. Bipedicle mucoperiosteal flaps were created on both sides of the cleft, which are approximated to cover the oral surface of the cleft (Figure 2).

In two-flap palatoplasty, an incision was made medial to dentition along the edge of the palate, starting anteriorly and continuing to the maxillary tuberosity and continuing onto the cleft soft palate and beyond the uvula onto the anterior tonsillar pillar. Mucoperiosteal flaps were elevated at the lateral and cleft edges to ensure adequate mucosa availability for nasal lining closure. Abnormal velar muscle attachments were dissected from the posterior edge of the hard palate and the nasal lining. Wound closure was initiated with the nasal lining, and muscles were then closed as a separate layer.

During Surgery

Before surgery, after the Dingman mouth gag has been inserted, the length of the soft palate is measured directly between the posterior notch of the hard palate and the tip of the uvula. After surgery, the length between the same points was measured again before the removal of the mouth gag. Post-surgery length from the same points was measured before the removal of the mouth gag.

Postoperative

Patients were put on injectable antibiotics, an anti-histaminic, and analgesics in the post-operative period for one day and were admitted for 2-3 days post-surgery. They were shifted to oral antibiotics and analgesics the next day and continued for 3 days. The patients' families were instructed to take adequate precautions. Immediately after surgery, the patients were assessed for respiratory distress, bleeding, and dehiscence of the wound, if present.

After Discharge

The patients were followed up at 2-weeks, 1-month, 3-months intervals. The patient and/or their families were interviewed for a history of leakage of liquids or semisolid foods through the nose at each follow-up. A careful visual inspection of the original line of cleft for oro-nasal fistulas was carried out using a hand-held torch.

Data collection and statistical analysis

The collected data was tabulated in Microsoft Excel (Microsoft Corporation, Redmond, USA). Analysis was carried out using Statistical Package for Social Sciences(SPSS) version 17.0 (SPSS Inc., Chicago, USA). Categorical data will be presented as percentages (%). Normally distributed data were presented as means and standard deviation, or 95% confidence intervals (CI). A paired t-test was performed to compare paired samples in the quantitative variables observed on Day 2 and Day 30.

## Results

Fifty patients were recruited in the current study and divided into two groups for comparison between Furlow’s and straight-line palatoplasty. The mean age of the patients enrolled was 0.93+ 0.21 years and 0.97+ 0.25 years for the Furlow’s and the straight-line groups, respectively (Table 1), with 54% of patients less than 12 months of age. The majority of patients were female (54%), with Furlow’s palatoplasty being the main procedure, while it was vice versa in males. 86% of the total patients were with incomplete cleft palate (Group II) with 84% and 88% of patients in each group being operated with Furlow’s palatoplasty and straight-line intravelar veloplasty, respectively (Table 1). Only 14% of study participants were found to have a complete cleft of the secondary palate (Group I+II) on pre-operative diagnosis.

**Table 1:** Demographic and pre-operative characteristics of patients undergoing corrective surgery for cleft lip and palate.

Variables	Furlow's Palatoplasty	Straight-line Intravelar veloplasty	Test of significance (chi-square)	p-value
Age (Mean)	0.93+ 0.21 years	0.97+ 0.25 years	0.148	>0.05
Sex (n)			0.725	>0.05
Males	10 (40%)	13 (52%)		
Females	15 (60%)	12 (48%)		
Complete Cleft of Secondary Palate (Group I+II)	4 (16%)	3 (12%)	0.1725	>0.05
Incomplete Cleft Palate (Group II)	21 (84%)	22 (88%)		

Table 2 shows the width of the clefts observed in the two groups before surgery. No significant difference was observed between the widths of the clefts in the two groups. 24% of the total patients presented with a 10 mm cleft size, followed by 9 mm (18%) and 8 mm (18%) cleft sizes.

**Table 2:** Width of the clefts observed in the two study groups.

Cleft Width (in mms)	Furlow's Palatoplasty	Straight-line intravelar veloplasty	Total	Test of significance (chi-square)	p-value
7	1 (4.0%)	0	1 (2.0%)	6.22	>0.05
8	5 (20.0%)	4 (16.0%)	9 (18.0%)		
9	6 (24.0%)	3 (12.0%)	9 (18.0%)		
10	6 (24.0%)	6 (24.0%)	12 (24.0%)		
11	4 (16.0%)	5 (20.0%)	9 (18.0%)		
12	1 (4.0%)	5 (20.0%)	6 (12.0%)		
13	2 (8.0%)	1 (4.0%)	3 (12.0%)		
16	0	1 (4.0%)	1 (2.0%)		

We recorded the duration of individual surgical procedures (Table 3). There was a significant difference between the mean duration of Furlow’s palatoplasty, which was around 81.2+8.2 minutes, and the mean duration of straight-line intravelar veloplasty, which took around 47.5+4.1 minutes, suggesting that straight-line intravelar veloplasty takes less time as compared to Furlow’s palatoplasty.

**Table 3:** Durations of surgeries in the two groups amongst the study participants.

	Furlow's Palatoplasty	Straight-line intravelar veloplasty	Test of significance (chi-square)	p-value
Less than 1 hour	0	25 (100%)	50	<0.05
1-1.5 hours	23 (92.0%)	0		
>1.5 hours	2 (8.0%)	0		

Both groups of patients were assessed for immediate complications post-surgery and were followed up at various time points (Table 4). Bleeding (8%) and dehiscence (4%) of wounds were immediate post-operative complications found in the straight-line group. Bleeding was also present in the Furlow's group (4%).

**Table 4:** Post-operative characteristics in the patients.

	Furlow's Palatoplasty	Straight-line intravelar veloplasty	Test of significance (chi-square)	p-value
Immediate Complications			1.87	>0.05
Bleeding	1 (4.0%)	2 (8.0%)		
Dehiscence	0	1 (4.0%)		
Wound at 2-Weeks Follow-up			1.49	>0.05
None	24 (96.0%)	22 (88.0%)		
Dehiscence	1 (4.0%)	3 (12.0%)		
Wound at 1-month Follow-up			1.54	>0.05
Fistula	1 (4.0%)	3 (12.0%)		
Minimal nasal regurgitation of liquids	1 (4.0%)	2 (8.0%)		
Wound at 3-month Follow up			1.54	>0.05
None	23 (92.0%)	20 (80.0%)		
Fistula	1 (4.0%)	3 (12.0%)		

At 2 weeks follow-up, about 12% of patients in the straight-line group had wound dehiscence, while in the Furlow's group, it was only 4% (p-value>0.05). At 1 month follow-up, wound status was assessed: 86% of patients had no post-operative complications, while in the straight-line group, about 12% of patients had developed with fistula, and 8% had minimal nasal regurgitation of liquids. In the Furlow's group, fistula and minimal nasal regurgitation of liquids were found in 4% of study participants for each complication.

**Figure 3:**
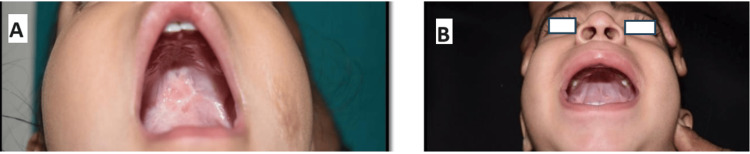
Representative images showing wound status at 3 months follow-up after cleft repair. A) Furlow’s Z-plasty; B) straight-line intravelar veloplasty.

Similar results were observed at 3 months follow-up (Figure 3) with 12% and 4% of the patients having developed fistula in the straight-line group and the Furlow's group, respectively. Follow-up results suggest that Furlow’s palatoplasty has a lower chance of developing fistulas.

**Figure 4:**
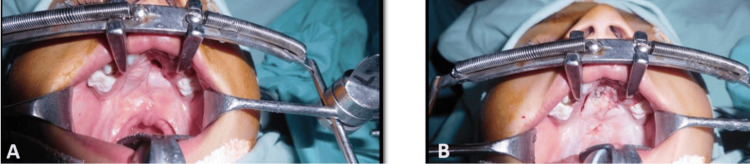
Representative images showing secondary surgery for post-operative fistula repair. A) Presence of fistula after straight-line intravelar veloplasty; B) fistula repair by local flap.

Table 5 shows palatal fistula characteristics in the patients subjected to two different surgical techniques observed at 3 months. Only 8% of study participants presented with palatal fistula at 3 months follow-up period. The incidence of palatal fistula was higher among participants of the straight-line group as compared to Furlow's palatoplasty. The location of the fistula was identified, and was observed that about 4% of fistulas were present in the anterior palate while the other 8% of fistulas were present at a junction in the straight-line group. 92% of study participants presented no fistula at 3 months follow-up period. The size of the fistula in the Furlow's group was observed to be 4 mm, while it was more than 4mm in the straight-line group. Secondary surgery for the post-operative fistula was fistula repair and secondary suturing, which were separately performed in one patient each in the straight-line group (Figure 4).

**Table 5:** Characteristics of fistula developed 3 months post-surgery in patients.

Variable	Furlow's Palatoplasty	Straight-line intravelar veloplasty	Test of significance (chi-square)	p-value
Incidence of Palatal fistula			1.09	>0.05
No	24 (96.0%)	22 (88.0%)		
Yes	1 (4.0%)	3 (12.0%)		
Location of Fistula			2.09	>0.05
Anterior	0	1 (4.0)		
Junctional	1(4.0%)	2 (8.0%)		
Size of Fistula				
4 mm	1 (100%)	0		
5 mm	0	2 (66.7%)		
8 mm	0	1 (33.3%)		
Secondary Surgery				
Fistula repair	0	1 (50.0%)		
Secondary Suturing	0	1(50.0%)		

Table 6 shows the lengthening of the soft palate by the two procedures. Mean soft palate length immediately after surgery was 34.6 mm and 30.6 mm in the Furlow’s and straight-line groups, respectively, while intra-operative elongation in the two groups was 6.44 mm and 1.64 mm, respectively, and was not statistically significant.

**Table 6:** Comparison of soft palate lengthening by Furlow’s palatoplasty and straight-line intravelar veloplasty.

Surgical Procedure	Soft Palate Lengthening (mm)			
-	Pre-operative	Post-operative	Intra-operative Elongation	p-value
Furlow’s Palatoplasty	28.2 ± 1.38	34.6 ± 1.22	6.44 ± 0.768	0.229
Straight-line intravelar veloplasty	29.04 ± 1.27	30.68 ± 1.49	1.64 ± 0.952	

## Discussion

There are various techniques for the correction of cleft palate. All these techniques aim at the anatomical closure of the palate, avoiding fistulas, providing a competent velum for adequate speech, and allowing harmonious facial growth. Refinements in the basic principles of repair and attention to anatomical restoration of the abnormally directed velar muscles have improved functional outcomes over the years. Currently, the techniques commonly adopted by cleft surgeons for the repair of palatal clefts include straight-line intravelar veloplasty and Furlow’s Z-palatoplasty.

The present study was undertaken to compare the outcomes of the Furlow's double-opposing Z-plasty and the straight-line Straight-line intravelar veloplasty techniques. There is debate among cleft surgeons on whether double-opposing Z-palatoplasty improves speech. Some argue it works by lengthening the palate and redirecting/overlapping levator muscles, While others raise concerns about an increased risk of palatal fistulas, particularly in wider clefts​​​​​​** **[8].

Most cleft surgeons focus on the type of repair to be performed in a period somewhere between 9 and 18 months of age as they are of the view that earlier repair may reduce the incidence of fistula. Rohrich et al. reported on a longitudinal cohort of patients in whom a two-stage palate repair was performed; those whose hard palate closure was performed at 10 months of age had a 5% fistula rate compared with a fistula rate of 35% when palate repair was delayed until 48 months [9]. In the present study also, the majority of the patients (72%) were below 12 months of age, and hence, the rate of fistula formation is reported to be less (Table 4).

Fistula rates following primary palatoplasty have historically been reported at over 60% and in the recent literature have ranged from 2.4% to 35% [10]. Multiple factors contribute to the development of palate fistulas, such as cleft width, the surgeon’s experience, mucosal tearing, large tissue displacement and creation of dead spaces, post-operative haemorrhage, etc. [11]. Criticisms of the Furlow's method have included higher fistula rates when double opposing Z-plasty is used in wider clefts and without relaxing incisions [12]. The present study has reported a difference in fistula rates between the Furlow's and straight-line intravelar veloplasty, i.e., 4% and 12%, respectively, although non-significant, which may be attributed to the small sample size. Our results were supported by a study by Gunther et al. which showed similar results [13]. A systemic review by Timbang et al. also reported no statistically significant difference in fistula rates between Furlow's and straight-line repair (7.87% and 9.81%, respectively) [12]. However, in each group, the fistula rate was significantly higher in unilateral cleft lips and palates compared to isolated clefts.

Cohen et al. reported a 23% fistula rate in 129 patients, with 43% fistula following Veau-Wardill-Kilner (VWK) procedures, 22% following straight-line procedures, and 10% following Furlow's repairs [14]. Chan et al. demonstrated that the frequency of palatal fistula after palatoplasty was 11.6% after straight-line repair and 5.1% after Furlow’s palatoplasty [15]. Arantes et al. also reported similar results, with fistula rates of 22.7% and 18.2% after straight-line intravelar veloplasty and Furlow's Z-plasty, respectively [16]. Grobbelaar et al. also showed that Fistula rates were higher among the straight-line and the VWK groups compared to the Furlow's group [17]. In contrast to our study's results, Williams et al. showed a significantly higher incidence of fistula in the Furlow's group as compared to the straight-line group [18].

In the present study, in the Furlow's palatoplasty group, palatal fistula occurred in the junctional (4%) region while in the straight-line intravelar veloplasty group, fistulas occurred in the anterior (4%) as well as the junctional region (8%). In a study done by Ravishankar, in the Furlow's group, three children developed fistulae at the junction of the soft and hard palates, giving an incidence of 9.09% [19]. In a systemic review done by Timbang et al., in the Furlow's group, most of the fistulae were located in the anterior hard palate, followed by junctional fistulae [12]. This finding is similar to the distribution reported by Smith et al. in a retrospective review of 641 cleft palates repaired by different techniques [20].

Regarding extension of the fistulae, in the present study, in the Furlow's group, one patient had small fistulae (4 mm), while in the straight-line group, two patients (66.7%) had medium-sized fistulae (5 mm), and one patient (33.3%) had a large fistula (8 mm). A study by Spauwen et al. also showed contrasting results as two palatal fistulae at the junction of the hard and soft palate with diameters of 2 mm and 3 mm, respectively, occurred in the Furlow's group (out of 10 surgeries done) and none in the straight-line group (out of 10 surgeries done) [21]. A study by Bosi et al. reported that seven patients (70%) had small fistulae (< 3 mm), three (30%) had medium-sized fistulae (3-5 mm), and no patients had a large fistula after straight-line intravelar veloplasty [22].

In the present study, straight-line intravelar veloplasty took less than 60 mins to get over as compared to Furlow’s palatoplasty, which took more than 60 minutes. The findings were similar to the study done by Ahmed and Kadah. In their study, the mean operative time was significantly shorter in the straight-line group (75±13, range 55-95 mins) as compared to the Furlow's group (84.3±8.2, range 70-100 mins) [23]. Sender and Sykes also reported a longer duration to complete Furlow's Z-plasty, with more loss of blood; however, in the end, this technique improves the palatal function as compared to classical palatoplasty techniques [24]. Ono et al. also showed that the time taken for the Furlow’s technique (83.9±14.9 mins) is longer as compared to other techniques [25]. However, a study by Brothers et al. showed no difference in intra-operative time (1.9 hours) between Furlow’s palatoplasty and VWK palatoplasty[26].

Immediate post-operative complications observed most frequently after cleft palate repair were hemorrhage/bleeding, respiratory obstruction, hanging palate, and dehiscence of the repair. In the present study, bleeding contributed to 8% of immediate post-operative complications in the straight-line group and 4% of the Furlow's palatoplasty group. Dehiscence of the wound was presented by the straight-line group(4%) only. While after a follow-up of 2 weeks, dehiscence of the wound was presented by 12% of cases in the straight-line group and 4% of the Furlow's group. Dubey et al. reported wound dehiscence in the straight-line group to be 13.33% of patients while in Furlow’s group, 6.66% of patients had wound dehiscence at the posterior nasal spine (PNS)region. Nasal regurgitation was not found in either of the groups [27].

In a study done by Bosi et al., bleeding was an immediate post-operative complication, found in 1.33% of patients after straight-line intravelar veloplasty. While dehiscence was present in 5% of patients as a late complication after the surgery [22]. Yu et al. also showed dehiscence as a complication in the straight-line group while none of the cases in the Furlow's group showed dehiscence of wound [28]. While Ono et al. reported that immediate post-operative bleeding is less with Furlow’s technique [25]). In a study done by Van Lierde et al., 5% of patients in the VWK palatoplasty group showed bleeding on re-exploration while none of the patients in the Furlow's group showed bleeding post-operatively [29].

In the present study, 4% of patients in the Furlow’s group showed nasal regurgitation while in the straight-line group, 8% of patients showed nasal regurgitation. Results are in contrast to the study done by Dubey et al., in their study nasal regurgitation was not found in either group [27].

In the present study, no secondary surgery was performed in the Furlow’s group at 3 months follow-up as there is one fistula in the group that needs secondary surgery after six months. In the straight-line group, local flap was performed in one patient with an anterior palatal fistula and secondary suturing was done for another patient. While in another patient with a palatal fistula, no secondary procedure was performed in the straight-line group as the patient had no symptoms.

In the present study, differences in soft palate lengthening by two procedures were compared and we did not observe a statistically significant difference in intra-operative elongation, however, the Furlow’s group had higher elongation as compared to the straight-line group which is of clinical importance. However, in a study conducted by Bae et al. in 2001, no significant differences were reported among the three palatoplasty techniques they compared [30].

Limitations of study

This is a single-center study, but the patients were operated on by both senior and junior surgeons randomly. So results might vary due to experience. Speech was not assessed as it required longer follow-up. Simple randomization was used, which leads to an imbalance in results.

## Conclusions

We analysed two different techniques of cleft palate repair - Furlow’s and straight-line intravelar veloplasty, and the results suggest that Furlow's Z-plasty is a more effective surgical procedure as it showed low immediate post-operative complications, less incidence of fistula development, and higher intra-operative soft-palate lengthening. However, some variations in the results are seen due to the cleft size, such as wider clefts, and the experience of surgeons. While initially, straight-line procedures took less intra-operative time than the Furlow's technique, particularly among junior surgeons who were less exposed to it, eventually the intra-operative time for Furlow's technique also decreased after more exposure to similar cases*.* The evaluation of the speech was not done because it necessitates a comprehensive approach, including serial assessments, extended follow-up, implementation of speech therapy, and consideration of minimally invasive procedures.

## References

[1] Mossey P, Little J (2009). Addressing the challenges of cleft lip and palate research in India. Indian J Plast Surg.

[2] Mossey PA, Modell B (2012). Epidemiology of oral clefts 2012: an international perspective. Front Oral Biol.

[3] Flint PW, Haughey BH, Robbins KT (2014). Cummings Otolaryngology: Head and Neck Surgery. https://www.asia.elsevierhealth.com/cummings-otolaryngology-9780323611794.html.

[4] Trier WC, Dreyer TM (1984). Primary von Langenbeck palatoplasty with levator reconstruction: rationale and technique. Cleft Palate J.

[5] Heliövaara A, Rintala A, Ranta R (1993). One-stage closure of isolated cleft palate with the Veau-Wardill-Kilner V to Y pushback procedure or the Cronin modification. I. Comparison of operative results. Scand J Plast Reconstr Surg Hand Surg.

[6] Kirschner RE, Wang P, Jawad AF (1999). Cleft-palate repair by modified Furlow double-opposing Z-plasty: the Children's Hospital of Philadelphia experience. Plast Reconstr Surg.

[7] Katzel EB, Basile P, Koltz PF, Marcus JR, Girotto JA (2009). Current surgical practices in cleft care: cleft palate repair techniques and postoperative care. Plast Reconstr Surg.

[8] Li F, Wang HT, Chen YY, Wu WL, Liu JY, Hao JS, Luo DY (2017). Cleft relapse and oronasal fistula after Furlow palatoplasty in infants with cleft palate: incidence and risk factors. Int J Oral Maxillofac Surg.

[9] Rohrich RJ, Rowsell AR, Johns DF (1996). Timing of hard palatal closure: a critical long-term analysis. Plast Reconstr Surg.

[10] Bykowski MR, Naran S, Winger DG, Losee JE (2015). The rate of oronasal fistula following primary cleft palate surgery: a meta-analysis. Cleft Palate Craniofac J.

[11] Saothonglang K, Punyavong P, Winaikosol K, Jenwitheesuk K, Surakunprapha P (2021). Risk factors of fistula following primary palatoplasty. J Craniofac Surg.

[12] Timbang MR, Gharb BB, Rampazzo A, Papay F, Zins J, Doumit G (2014). A systematic review comparing Furlow double-opposing Z-plasty and straight-line intravelar veloplasty methods of cleft palate repair. Plast Reconstr Surg.

[13] Gunther E, Wisser JR, Cohen MA, Brown AS (1998). Palatoplasty: Furlow's double reversing Z-plasty versus intravelar veloplasty. Cleft Palate Craniofac J.

[14] Cohen SR, Kalinowski J, LaRossa D, Randall P (1991). Cleft palate fistulas: a multivariate statistical analysis of prevalence, etiology, and surgical management. Plast Reconst Surg.

[15] Chan EKW, Lee KH, Tsui BSY (2014). From von Langenbeck to Furlow palatoplasty: a 16‐year review of cleft palate repair. Surg Prac.

[16] Arantes HL, Zampar A, Oliveira Junior FCd, Rosique M, Rosique RG, Leal WA (2008). Fístulas e deiscências em palatoplastia primária: uma experiência institucional. Rev Bras Cir Plást.

[17] Grobbelaar AO, Hudson DA, Fernandes DB, Lentin R (1995). Speech results after repair of the cleft soft palate. Plast Reconstr Surg.

[18] Williams WN, Seagle MB, Pegoraro-Krook MI (2011). Prospective clinical trial comparing outcome measures between Furlow and von Langenbeck palatoplasties for UCLP. Ann Plast Surg.

[19] Ravishanker R (2006). Furlow's palatoplasty for cleft palate repair. Med J Armed Forces India.

[20] Smith DM, Vecchione L, Jiang S (2007). The Pittsburgh Fistula Classification System: a standardized scheme for the description of palatal fistulas. Cleft Palate Craniofac J.

[21] Spauwen PH, Goorhuis-Brouwer SM, Schutte HK (1992). Cleft palate repair: Furlow versus von Langenbeck. J Cranio-Maxillofac Surg.

[22] Bosi V, Brandão G, Yamashita R (2001). Speech resonance and surgical complications after primary palatoplasty with intravelar veloplasty in patients with cleft lip and palate. Rev Bras Cirurg Plást.

[23] Ahmed M, Kadah SM (2011). Furlow's palatoplasty is a safe competent procedure for isolated cleft palate repair. Kasr El Aini J Surg.

[24] Senders CW, Sykes JM (1995). Modifications of the Furlow palatoplasty (six- and seven-flap palatoplasties). Arch Otolaryngol Head Neck Surg.

[25] Ono K, Ohashi Y, Kannari Y, Isono S (1995). Cleft palate repair by Furlow double opposing Z-plasty II. Speech results and effects on maxillary growth. Japan J Oral Maxillofac Surg.

[26] Brothers DB, Dalston RW, Peterson HD, Lawrence WT (1995). Comparison of the Furlow double-opposing Z-palatoplasty with the Wardill-Kilner procedure for isolated clefts of the soft palate. Plast Reconstr Surg.

[27] Dubey P, Kumar S, Gupta R, Bansal V, Mowar AM, Khare G (2015). Comparative evaluation of modified furlow palatoplasty and intravelar veloplasty in cleft palate repair. Am J Oral Maxillofac Surg.

[28] Yu CC, Chen PK, Chen YR (2001). Comparison of speech results after Furlow palatoplasty and von Langenbeck palatoplasty in incomplete cleft of the secondary palate. Chang Gung Med J.

[29] Van Lierde KM, Monstrey S, Bonte K, Van Cauwenberge P, Vinck B (2004). The long-term speech outcome in Flemish young adults after two different types of palatoplasty. Int J Pediatr Otorhinolaryngol.

[30] Bae YC, Kim JH, Lee J, Hwang SM, Kim SS (2002). Comparative study of the extent of palatal lengthening by different methods. Ann Plast Surg.

